# Association between Body Mass Index and Short-Term Clinical Outcomes in Critically Ill Patients with Sepsis: A Real-World Study

**DOI:** 10.1155/2020/5781913

**Published:** 2020-10-15

**Authors:** Shan Lin, Shanhui Ge, Wanmei He, Mian Zeng

**Affiliations:** Department of Medical Intensive Care Unit, The First Affiliated Hospital of Sun Yat-sen University, No. 58 Zhongshan Road 2, Guangzhou, Guangdong 510080, China

## Abstract

**Background:**

Obesity is now recognized as one of the major public health threats, especially for patients with a critical illness. However, studies regarding whether and how body mass index (BMI) affects clinical outcomes in patients with sepsis are still scarce and controversial. The aim of our study was to determine the effect of BMI on critically ill patients with sepsis.

**Materials and Methods:**

We performed this study using data from the Medical Information Center for Intensive Care III database. A multivariate Cox regression model was used to assess the independent association of BMI with the primary outcome.

**Results:**

A total of 7,967 patients were enrolled in this study. Firstly, we found that the 28-day mortality was reduced by 22% (HR = 0.78, 95% CI 0.69–0.88) and 13% (HR = 0.87, 95% CI 0.78–0.98) for obese and overweight compared to normal weight, respectively. Subsequently, a U-shaped association of BMI with 28-day mortality was observed in sepsis patients, with the lowest 28-day mortality at the BMI range of 30–40 kg/m^2^. Finally, significant interactions were observed only for sex (*P* = 0.0071). Male patients with a BMI of 25-30 kg/m^2^ (HR = 0.74, 95% CI 0.63–0.86) and 30-40 kg/m^2^ (HR = 0.63, 95% CI 0.53–0.76) had a significantly lower risk of 28-day mortality.

**Conclusions:**

A U-shaped association of BMI with 28-day mortality in critically ill sepsis patients was found, with the lowest 28-day mortality at a BMI range of 30–40 kg/m^2^. Notably, male patients were protected by a higher BMI more effectively than female patients as males had a significantly lower mortality risk.

## 1. Introduction

With the rapidly increasing prevalence of obesity, healthcare professionals are faced with an increasing number of obese patients with a critical illness [1, 2]. Obesity is currently one of the major public health threats, which affects almost all physiological functions. Multiple investigators hold that this phenomenon of increased BMI or obesity will develop a detrimental effect in morbidity and mortality from obesity-related diseases, such as diabetes mellitus, cardiovascular disease, poor mental health, musculoskeletal diseases, and several types of cancer [3–6]. However, there are controversies over the evidence available for the mortality caused by overweight and obesity. Various studies have shown a U-shaped association between BMI and population mortality, with the highest risk of death in underweight and overweight populations [7–11].

Sepsis is an acute life-threatening organ dysfunction secondary to infection, and morbidity and mortality rates remain high [12–14]. Currently, it has yet to be established whether the mortality rate among sepsis patients is truly related to their BMI and, if so, to what extent. Accordingly, it is well worth exploring the correlation between BMI and the short-term prognosis of patients with sepsis. Given this, we aimed to assess the impact of BMI on critically ill patients with sepsis upon admission to the intensive care unit (ICU).

## 2. Materials and Methods

### 2.1. Patient Data

We conducted this study in the Medical Information Center for Intensive Care (MIMIC-III) database [15]. The Institutional Review Boards of the Beth Israel Deaconess Medical Center and MIT-affiliated institutions have approved access to this database (record ID: 33460949). Patient consent was waived due to the retrospective study and the anonymity of the data.

All patients' data (age ≥ 18 years) with a diagnosis of sepsis-3 based on a sequential organ failure assessment (SOFA) score of ≥2 and suspected infection within the first day of ICU admission during the period 2002 to 2012 were included [12, 16]. BMI was calculated by weight and height measured on the first day of ICU admission. Patients with missing weight or height parameters and a BMI of less than 10 kg/m^2^ or more than 60 kg/m^2^ were excluded. In compliance with the World Health Organization BMI classification, patients were categorized as follows: the underweight group (BMI < 18.5 kg/m^2^), the normal group (BMI ≥ 18.5, <25 kg/m^2^), the overweight group (BMI ≥ 25, <30 kg/m^2^), and the obese group (BMI ≥ 30 kg/m^2^). As mentioned in our previous study, structured query language (SQL) was used to extract data in the software Navicat Premium [17]. The code and website used for the MIMIC-III database are publicly available [18].

### 2.2. Outcomes

The primary clinical outcome was 28-day mortality, and the secondary outcome was ICU mortality.

### 2.3. Statistical Analysis

Data were expressed as median (Q1–Q3) or mean ± standard deviation (SD) for continuous variables and as numbers and percentages for categorical variables. We used the Chi-square test (for categorical variables) and one-way analysis of variance (for continuous variables) to compare differences in patient characteristics between groups. To begin, we utilized Cox regression and logistic regression models to investigate the independent roles of BMI categories in relation to the primary and secondary outcomes. Initially, the Cox regression model was adjusted for demographic factors, treatment, and disease severity scores. Next, we replaced the Elixhauser Comorbidity Index (SID30) [19] score with specific diseases to more accurately assess the independent effect of BMI on outcomes.

Subsequently, to determine if there was a U-shaped association between 28-day mortality rate and BMI, nonlinear regression analyses using the Cox model were conducted to fit the clinical outcome. A two-piecewise linear regression model and a log-likelihood ratio test were used to screen for the presence of a threshold effect of 28-day mortality. The inflection points were shown using threshold effect analysis. The confidence interval of the inflection point was calculated through the bootstrap method [20]. Age- and sex-adjusted Kaplan-Meier's (K-M) curves were used to assess the effect of the new BMI category on 28-day mortality. Lastly, we also performed interaction tests and stratified analyses on variables such as age (<65 and ≥65 years), sex, and SOFA score (<5, 5-10, 10-15, and ≥15). All data were collected using EmpowerStats (R) (http://www.empowerstats.com), X & Y Solutions, Inc., Boston, Massachusetts, USA) and R (http://www.R-project.org, version 3.4.3) for analysis. A *P* value of less than 0.05 was considered significant.

## 3. Results

### 3.1. Characteristics of the Study Population

**Figure 1:**
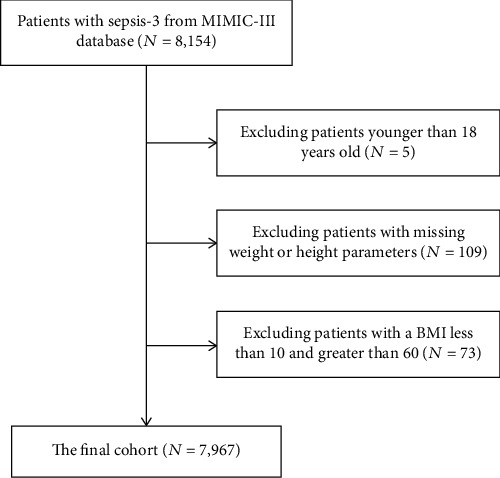
Flow chart for the selection of study participants.

Data of 7,967 patients were used in this study (Table 1 and Figure 1). The mean age of the study population was 65.92 ± 15.98 years, and the mean age of overweight and obese patients was markedly lower than that of normal and underweight patients (*P* < 0.001). Males accounted for 55.25% of all participants. The median SOFA scores were higher in overweight and obese patients than in normal and underweight patients (*P* < 0.001), while the median SID30 scores, however, were lower than those of normal and underweight patients. A higher proportion of obese patients required mechanical ventilation on the first day of ICU admission than in the remaining three groups. No statistically significant differences were observed in renal replacement therapy between BMI categories on the first day, whereas statistically significant differences were not observed among the groups requiring renal replacement therapy on the first day. Comparisons with the underweight, overweight, and obese groups using normal weight as a reference are placed in Tables S1–S3.

### 3.2. Clinical Outcomes of Participants

Overall, hospital mortality and ICU mortality were dramatically lower in the obese group than in the normal weight group (19.14% vs. 24.0%, *P* < 0.001 and 13.81% vs. 17.35%, *P* = 0.005) (Table 1). On day 28, 1,667 patients (20.92%) died after ICU admission. In addition, obese patients had a lower 28-day mortality rate in comparison to normal weight patients (17.49% vs. 23.76%, *P* < 0.001). However, compared with overweight, normal, and underweight patients, obese patients suffered longer lengths of ICU and hospital stay (*P* < 0.001).

### 3.3. Associations between BMI and Clinical Outcomes

The multivariate Cox regression analysis showed that overweight and obese patients were protected against 28-day mortality, with crude risk ratios of 0.87 (95% CI 0.77–0.98) and 0.71 (95% CI 0.63–0.80), respectively. After adjustment, we found that overweight and obese were still not risk factors, and the results were consistent and significant across the adjusted models (Table 2). In model II, the 28-day mortality was reduced by 22% (HR 0.78, 95% CI 0.69–0.88, *P* = 0.0001) and 13% (HR 0.87, 95% CI 0.78–0.98, *P* = 0.0263) for the obese and overweight groups compared to the normal weight group, respectively. Similar results were also found for ICU mortality (Table 2). In model II, the multivariate logistic regression analysis revealed that there was a relatively low risk of ICU mortality in the obese group (OR 0.77, 95% CI 0.65–0.91, *P* = 0.0023).

**Figure 2:**
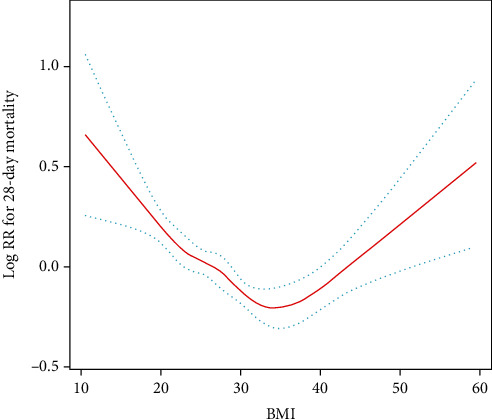
Association between BMI and 28-day mortality. Notes: adjusted by age, sex, SOFA, mechanical ventilation on the first day, renal replacement therapy on the first day, congestive heart failure, cardiac arrhythmias, valvular disease, peripheral vascular disease, hypertension, other neurological diseases, chronic pulmonary disease, liver disease, renal failure, AIDS, lymphoma, metastatic cancer, solid tumor, diabetes, fluid and electrolyte disorders, alcohol abuse, drug abuse, and depression.

Smooth curve fitting showed a U shape as the 28-day mortality rate decreased with increasing BMI before the inflection point (Figure 2). By using the threshold effect analysis, the inflection point was determined as 38.68 kg/m^2^ (95% CI 26.49–40.99 kg/m^2^) for the 28-day mortality rate. The threshold effect of BMI on the 28-day mortality group was significant after adjusting for potential confounders. The hazard ratio was 0.97 for BMI < 38.68 kg/m^2^ and 1.05 for BMI ≥ 38.68 kg/m^2^ for the 28-day mortality rate (Table 3). The log-likelihood ratio test was less than 0.001.

**Figure 3:**
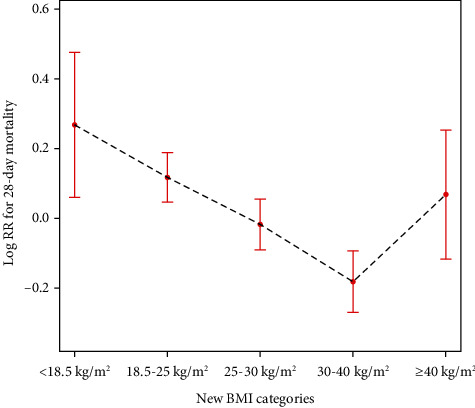
The 28-day mortality across fitted groups of new BMI categories. Notes: adjusted by age, sex, SOFA, mechanical ventilation on the first day, renal replacement therapy on the first day, congestive heart failure, cardiac arrhythmias, valvular disease, peripheral vascular disease, hypertension, other neurological diseases, chronic pulmonary disease, liver disease, renal failure, AIDS, lymphoma, metastatic cancer, solid tumor, diabetes, fluid and electrolyte disorders, alcohol abuse, drug abuse, and depression.

**Figure 4:**
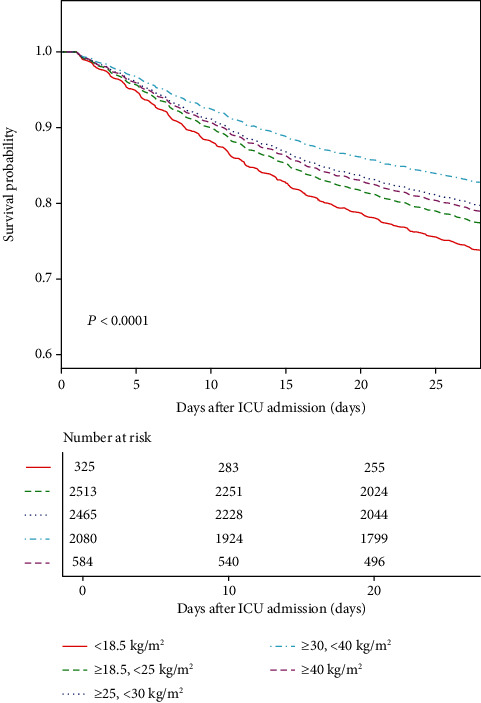
Kaplan-Meier's survival curve by new BMI categories in 28-day mortality adjusting age and sex.

Based on the 95% confidence interval of the inflection point calculated by the bootstrap method, we divided the BMI between 30 and 40 kg/m^2^ into a separate group; the 28-day mortality across fitted groups of new BMI categories is presented in Figure 3. The K-M curves showed that there were significant survival characteristics in patients with the BMI range of 30–40 kg/m^2^ after adjusting age and sex (log-rank test: *P* < 0.0001) (Figure 4).

In the stratified analysis, the association between the new BMI categories and the risk of 28-day mortality was similar for most strata (*P* > 0.05) (Table 4). A significant interaction was observed only for sex (*P* = 0.0071). Male patients were protected by a higher BMI more effectively than female patients as males had a significantly lower mortality risk. Male patients with a BMI of 25-30 kg/m^2^ and 30-40 kg/m^2^ had a significantly lower risk of 28-day mortality (HR 0.74, 95% CI 0.63–0.86, *P* = 0.0001 and HR 0.63, 95% CI 0.53–0.76, *P* < 0.0001, respectively). However, in the case of female patients, a 47% increased 28-day mortality was observed only in patients with a BMI < 18.5 kg/m^2^ (HR 1.47, 95% CI 1.09–2.00).

## 4. Discussion

In this study, we selected a specific study population with sepsis who was admitted to the ICU from a sizeable critical care database and explored the association between BMI and clinical outcomes. As a consequence, a U-shaped association was found between BMI and 28-day mortality in patients with sepsis, with the lowest 28-day mortality in BMI between 30 and 40 kg/m^2^.

The opinion that obesity can lead to multiple diseases due to a less healthy lifestyle is largely accepted. The condition of obese patients is almost not optimistic when seriously ill. In the general population, BMI itself can be a powerful predictor of the overall mortality rate when it is outside the range of the optimal BMI (23–25 kg/m^2^) [11, 21]. On the contrary, in patients who are overweight or obese, BMI and overall mortality have an inverse relationship, known as “obesity paradox” [22, 23]. A cohort study of 55,038 adult patients claimed that the short-term mortality rate was lower in patients with higher BMI after unadjusted and adjusted analyses [24]. Similarly, an observational cohort study showed a negative association between obesity and hospital mortality in critically ill patients [25]. Additionally, another meta-analysis revealed an inverse association between overweight (BMI between 25 and 29.9 kg/m^2^) or obesity (BMI between 30 and 39.9 kg/m^2^) and mortality in critically ill patients [26]. Similarly, in four other recent meta-analyses, obesity was found to be markedly associated with reduced mortality in critically ill patients [27–30].

In contrast to our findings and the results above, a retrospective study of 1,191 adults admitted with severe sepsis showed that the adjusted 28-day mortality rate was neither significantly increased nor decreased in obese or morbidly obese patients compared to that of normal weight patients [31]. A meta-analysis including 22 studies, with a total of 88,051 patients, indicated that obesity did not impact the mortality rate of patients admitted to the ICU [32]. Additionally, another meta-analysis conducted by Akinnusi involving 62,045 critically ill patients found that obesity was not correlated with an elevated risk of death in the ICUs. Nevertheless, the survival rate of obese patients with a BMI between 30 and 39.9 kg/m^2^ was improved, which was in accordance with the results of Oliveros et al. [33]. However, the meta-analysis of observational studies should be construed cautiously, and the evidence of association should not be mistaken for proof of causality. Importantly, these meta-analyses did not make any adjusting for disease severity or age. Furthermore, the BMIs were inaccurate since fluid overload led to pseudoobesity in certain cases, which ultimately affected the outcomes. Such inconsistencies were also observed in the retrospective studies. Finally, some studies reported losing a large amount of data, and heterogeneity could be found in data collection from included studies.

Hence, it is still unclear whether the obesity paradox exists as the underlying physiological mechanisms are not fully understood. Some evidence-based explanations are as follows: (a) A higher BMI means that a higher nutritional reserve is available, which is essential for surviving an acute life-threatening illness. (b) Adipose tissue regulates the inflammatory response by secreting anti-inflammatory mediators such as leptin, interleukin-10 (IL-10), and soluble tumor necrosis factor receptor 2 [34–36]. Earlier studies suggested that in critically ill patients with sepsis, lower levels of plasma IL-10 were associated with poor prognosis [37]. Similarly, serum leptin levels were significantly higher in sepsis survivors [38]. (c) High-density lipoproteins in obese patients can bind to bacterial endotoxins, which in turn is beneficial during sepsis [36]. (d) increased renin-angiotensin system activation may confer hemodynamic advantages in sepsis [39]. Nevertheless, these potential mechanisms are based on the guesswork of basic research. The beneficial effects of obesity cannot be explained yet as the determination of body fat distribution through BMI calculation is not possible.

There were also several limitations in our study. We tried to exclude the effects of “false obesity” due to interventions by extracting the first BMI after ICU admission. However, we still cannot determine whether the interventions that patients have received before ICU admission may affect BMI, such as intravenous infusions. Compared with the inclusion criteria by ICD-9 code in the previous study [40], we used the sepsis-3 diagnostic criteria to enroll the study population. Moreover, our study took into account the effect of comorbidities, and, to our knowledge, this is the first time a U-shaped relationship between BMI and 28-day mortality has been found. These findings were one strength of our research that was different from previous studies. But it cannot be ignored that we assumed a baseline SOFA score of 0 points prior to ICU admission and thus used a SOFA ≥ 2 points to include patients, which may result in overinclusion of patients; similarly, we did not consider biomarkers like PCT and interleukins, which would potentially have an impact on outcomes. Other limitations included selection bias in a retrospective study, a single-center design, and low external validity. Therefore, well-designed prospective multicenter studies are definitely required to further confirm our findings.

## 5. Conclusion

In this study, we found a U-shaped relationship between BMI and 28-day mortality in critically ill sepsis patients, with the lowest 28-day mortality at a BMI range of 30–40 kg/m^2^. Notably, male patients were protected by a higher BMI more effectively than female patients as males had a significantly lower mortality risk.

## Figures and Tables

**Table 1:** Characteristics of participants.

Parameters	All patients	Underweight (<18.5 kg/m^2^)	Normal (≥18.5, <25 kg/m^2^)	Overweight (≥25, <30 kg/m^2^)	Obese (≥30 kg/m^2^)	*P* value
*N*	7967	325	2513	2465	2664	
Age (years)	65.92 ± 15.98	67.02 ± 17.6	67.56 ± 16.88	66.51 ± 16.21	63.71 ± 14.36	<0.001
Sex, n(%)						<0.001
Male	4402 (55.25%)	155 (47.69%)	1363 (54.24%)	1486 (60.28%)	1398 (52.48%)	
Female	3565 (44.75%)	170 (52.31%)	1150 (45.76%)	979 (39.72%)	1266 (47.52%)	
SOFA	5.00 (3.00-8.00)	4.00 (3.00-8.00)	5.00 (3.00-8.00)	6.00 (4.00-8.00)	6.00 (4.00-8.00)	<0.001
Elixhauser Comorbidity Index (SID30)	19.00 (10.00-29.00)	21.00 (12.00-31.00)	20.00 (11.00-30.00)	20.00 (11.00-29.00)	18.00 (9.00-28.00)	<0.001
Mechanical ventilation on the first day	4918 (61.73%)	175 (53.85%)	1493 (59.41%)	1511 (61.30%)	1739 (65.28%)	<0.001
Renal replacement therapy on the first day	536 (6.73%)	18 (5.54%)	152 (6.05%)	169 (6.86%)	197 (7.39%)	0.209
Length of hospital stay (days)	14.46 (8.15-24.69)	11.86 (7.07-21.86)	13.66 (7.73-23.58)	14.64 (8.32-24.99)	15.51 (8.91-25.39)	<0.001
Length of ICU stay (days)	5.85 (2.80-12.04)	4.38 (2.22-8.99)	5.23 (2.53-11.16)	5.86 (2.86-11.92)	6.50 (3.05-13.30)	<0.001
Hospital mortality, *n* (%)	1723 (21.63%)	77 (23.69%)	603 (24.00%)	533 (21.62%)	510 (19.14%)	<0.001
ICU mortality, *n* (%)	1254 (15.74%)	53 (16.31%)	436 (17.35%)	397 (16.11%)	368 (13.81%)	0.005
28-day mortality, *n* (%)	1667 (20.92%)	89 (27.38%)	597 (23.76%)	515 (20.89%)	466 (17.49%)	<0.001
Comorbidities, *n* (%)						
Congestive heart failure	3194 (40.09%)	107 (32.92%)	972 (38.68%)	979 (39.72%)	1136 (42.64%)	<0.001
Cardiac arrhythmias	3047 (38.25%)	85 (26.15%)	989 (39.36%)	936 (37.97%)	1037 (38.93%)	<0.001
Valvular disease	1317 (16.53%)	49 (15.08%)	447 (17.79%)	403 (16.35%)	418 (15.69%)	0.187
Peripheral vascular disease	1133 (14.22%)	57 (17.54%)	387 (15.40%)	365 (14.81%)	324 (12.16%)	0.001
Hypertension	4326 (54.30%)	129 (39.69%)	1286 (51.17%)	1332 (54.04%)	1579 (59.27%)	<0.001
Other neurological disease	1130 (14.18%)	62 (19.08%)	387 (15.40%)	369 (14.97%)	312 (11.71%)	<0.001
Chronic pulmonary disease	1913 (24.01%)	102 (31.38%)	602 (23.96%)	529 (21.46%)	680 (25.53%)	<0.001
Liver disease	987 (12.39%)	39 (12.00%)	276 (10.98%)	310 (12.58%)	362 (13.59%)	0.041
Renal failure	1806 (22.67%)	67 (20.62%)	523 (20.81%)	588 (23.85%)	628 (23.57%)	0.030
AIDS	104 (1.31%)	7 (2.15%)	56 (2.23%)	28 (1.14%)	13 (0.49%)	<0.001
Lymphoma	216 (2.71%)	12 (3.69%)	68 (2.71%)	72 (2.92%)	64 (2.40%)	0.464
Metastatic cancer	436 (5.47%)	24 (7.38%)	172 (6.84%)	151 (6.13%)	89 (3.34%)	<0.001
Solid tumor	386 (4.84%)	29 (8.92%)	129 (5.13%)	112 (4.54%)	116 (4.35%)	0.003
Diabetes	2468 (30.98%)	45 (13.85%)	553 (22.01%)	713 (28.92%)	1157 (43.43%)	<0.001
Fluid and electrolyte disorders	3876 (48.65%)	163 (50.15%)	1221 (48.59%)	1201 (48.72%)	1291 (48.46%)	0.952
Alcohol abuse	625 (7.84%)	14 (4.31%)	196 (7.80%)	200 (8.11%)	215 (8.07%)	0.108
Drug abuse	291 (3.65%)	9 (2.77%)	117 (4.66%)	84 (3.41%)	81 (3.04%)	0.011
Depression	791 (9.93%)	35 (10.77%)	211 (8.40%)	245 (9.94%)	300 (11.26%)	0.007

Data are expressed as median (Q1–Q3) or mean ± standard deviation (SD) for continuous variables and as numbers and percentages for categorical variables. Chi-square tests (for categorical variables) and one-way analysis of variance (for continuous variables) were used to compare differences in patient characteristics between groups. Abbreviations: ICU—intensive care unit; SOFA—sequential organ failure assessment; AIDS—acquired immune deficiency syndrome.

**(a):** 

28-day mortality	Groups	HR (95% CI)	*P* value
Crude	Normal	1.0	—
	Underweight	1.17 (0.94-1.47)	0.1609
	Overweight	0.87 (0.77-0.98)	0.0175
	Obese	0.71 (0.63-0.80)	<0.0001
Model I	Normal	1.0	—
	Underweight	1.20 (0.96-1.50)	0.1117
	Overweight	0.86 (0.76-0.97)	0.0130
	Obese	0.75 (0.66-0.84)	<0.0001

Model II	Normal	1.0	—
	Underweight	1.16 (0.93-1.46)	0.1845
	Overweight	0.87 (0.78-0.98)	0.0263
	Obese	0.78 (0.69-0.88)	0.0001

**(b):** 

ICU mortality	Groups	OR (95% CI)	*P* value
Crude	Normal	1.0	—
	Underweight	0.93 (0.68-1.27)	0.6398
	Overweight	0.91 (0.79-1.06)	0.2397
	Obese	0.76 (0.66-0.89)	0.0005
Model I	Normal	1.0	—
	Underweight	1.01 (0.73-1.41)	0.9365
	Overweight	0.86 (0.73-1.01)	0.0578
	Obese	0.73 (0.62-0.85)	0.0001
Model II	Normal	1.0	—
	Underweight	0.97 (0.70-1.36)	0.8721
	Overweight	0.87 (0.74-1.02)	0.0902
	Obese	0.77 (0.65-0.91)	0.0023

Cox regression models and logistic regression models were used to examine the independent role of BMI category in relation to 28-day and ICU mortality. Model I was adjusted by age, sex, SOFA, Elixhauser Comorbidity Index (SID30), mechanical ventilation on the first day, and renal replacement therapy on the first day. Model II was adjusted by age, sex, SOFA, mechanical ventilation on the first day, renal replacement therapy on the first day, congestive heart failure, cardiac arrhythmias, valvular disease, peripheral vascular disease, hypertension, other neurological diseases, chronic pulmonary disease, liver disease, renal failure, AIDS, lymphoma, metastatic cancer, solid tumor, diabetes, fluid and electrolyte disorders, alcohol abuse, drug abuse, and depression. Abbreviations: HR—hazard ratio; OR—odds ratio; CI—confidence interval; SOFA—sequential organ failure assessment; AIDS—acquired immune deficiency syndrome.

**Table 3:** Threshold effect analysis of BMI and 28-day mortality.

Outcome: 28-year mortality			
Inflection point	HR	95% CI	*P* value
<38.68 kg/m^2^	0.97	0.96-0.98	<0.0001
≥38.68 kg/m^2^	1.05	1.03-1.08	<0.0001
The log-likelihood ratio test: *P* < 0.001			
The confidence interval of the inflection point: 26.49–40.99 kg/m^2^			

A two-piecewise linear regression model and a log-likelihood ratio test were used to explore for the presence of a threshold effect of 28-day mortality, and inflection points are shown using threshold effect analysis, with confidence interval of the inflection point calculated by the bootstrap method. Note: adjusted by age, sex, SOFA, mechanical ventilation on the first day, renal replacement therapy on the first day, congestive heart failure, cardiac arrhythmias, valvular disease, peripheral vascular disease, hypertension, other neurological diseases, chronic pulmonary disease, liver disease, renal failure, AIDS, lymphoma, metastatic cancer, solid tumor, diabetes, fluid and electrolyte disorders, alcohol abuse, drug abuse, and depression. Abbreviations: BMI—body mass index; HR—hazard ratio; CI—confidence interval.

**Table 4:** Effect size of new BMI categories on 28-day mortality rate in prespecified and exploratory subgroups in each subgroup.

Outcomes: 28-year mortality	Crude		Adjusted model		
BMI (kg/m^2^)	HR (95% CI)	*P* value	HR (95% CI)	*P* value	*P* interaction
Age (years): <65					0.6539
≥18.5, <25	1.0	—	1.0	—	
<18.5	1.09 (0.71-1.67)	0.6932	1.20 (0.78-1.85)	0.4028	
≥25, <30	0.88 (0.71-1.10)	0.2646	0.87 (0.69-1.08)	0.2014	
≥30, <40	0.82 (0.65-1.03)	0.0857	0.78 (0.62-0.99)	0.0410	
≥40	0.76 (0.56-1.03)	0.0773	0.91 (0.66-1.25)	0.5455	
Age (years): ≥65					
≥18.5, <25	1.0	—	1.0	—	
<18.5	1.20 (0.92-1.55)	0.1764	1.16 (0.89-1.51) 0.87	0.2775	
≥25, <30	0.87 (0.75-1.00)	0.0456	(0.75-1.00)	0.0457	
≥30, <40	0.67 (0.57-0.78)	<0.0001	0.67 (0.56-0.79)	<0.0001	
≥40	0.89 (0.68-1.17)	0.4194	0.85 (0.64-1.13)	0.2604	
Sex: male					0.0071
≥18.5, <25	1.0	—	1.0	—	
<18.5	0.92 (0.66-1.28)	0.6098	0.90 (0.64-1.27)	0.5562	
≥25, <30	0.77 (0.66-0.89)	0.0007	0.74 (0.63-0.86)	0.0001	
≥30, <40	0.59 (0.50-0.71)	<0.0001	0.63 (0.53-0.76)	<0.0001	
≥40	0.69 (0.52-0.92)	0.0119	0.86 (0.64-1.16)	0.3333	
Sex: female					
≥18.5, <25	1.0	—	1.0	—	
<18.5	1.47 (1.09-1.99)	0.0123	1.47 (1.09-2.00)	0.0128	
≥25, <30	1.02 (0.85-1.23)	0.8039	1.13 (0.94-1.35)	0.2001	
≥30, <40	0.84 (0.69-1.02)	0.0715	0.92 (0.76-1.13)	0.4268	
≥40	0.75 (0.57-0.99)	0.0438	1.05 (0.79-1.41)	0.7243	
Mechanical ventilation on first day: no					0.2258
≥18.5, <25	1.0	—	1.0	—	
<18.5	0.97 (0.68-1.39)	0.8722	1.01 (0.70-1.46) 0.92	0.9497	
≥25, <30	0.89 (0.73-1.07)	0.2096	(0.76-1.12)	0.3921	
≥30, <40	0.63 (0.50-0.79)	<0.0001	0.66 (0.52-0.83)	0.0005	
≥40	0.83 (0.59-1.18)	0.3042	1.08 (0.76-1.55)	0.6638	
Mechanical ventilation on first day: yes					
≥18.5, <25	1.0	—	1.0	—	
<18.5	1.33 (1.00-1.77)	0.0465	1.27 (0.95-1.69)	0.1054	
≥25, <30	0.85 (0.74-0.99)	0.0391	0.85 (0.73-0.99)	0.0390	
≥30, <40	0.72 (0.62-0.85)	<0.0001	0.79 (0.67-0.93)	0.0045	
≥40	0.65 (0.51-0.83)	0.0006	0.84 (0.65-1.08)	0.1728	
Renal replacement therapy on first day: no					0.2344
≥18.5, <25	1.0	—	1.0	—	
<18.5	1.20 (0.96-1.51)	0.1153	1.22 (0.97-1.54)	0.0876	
≥25, <30	0.86 (0.76-0.97)	0.0144	0.88 (0.78-1.00)	0.0460	
≥30, <40	0.70 (0.61-0.80)	<0.00010.0035	0.76 (0.66-0.88)	0.0002	
≥40	0.73 (0.59-0.90)		0.97 (0.78-1.20)	0.7656	
Renal replacement therapy on first day: yes					
≥18.5, <25	1.0	—	1.0	—	
<18.5	0.71 (0.28-1.78)	0.4650	0.74 (0.28-1.93)	0.5383	
≥25, <30	0.93 (0.64-1.36)	0.7092	0.81 (0.55-1.21)	0.3076	
≥30, <40	0.61 (0.40-0.94)	0.0258	0.63 (0.40-0.99)	0.0433	
≥40	0.45 (0.22-0.92)	0.0284	0.50 (0.24-1.05)	0.0678	
SOFA: <5					0.7859
≥18.5, <25	1.0	—	1.0	—	
<18.5	1.22 (0.85-1.75)	0.2771	1.14 (0.79-1.64)	0.4928	
≥25, <30	0.81 (0.65-1.02)	0.0693	0.87 (0.70-1.09)	0.2383	
≥30, <40	0.54 (0.42-0.71)	<0.0001	0.68 (0.52-0.90)	0.0066	
≥40	0.53 (0.34-0.83)	0.0055	0.85 (0.53-1.36)	0.5003	
SOFA: ≥5, <10					
≥18.5, <25	1.0	—	1.0	—	
<18.5	1.29 (0.92-1.82)	0.1359	1.18 (0.84-1.66)	0.3522	
≥25, <30	0.79 (0.66-0.95)	0.0101	0.81 (0.67-0.97)	0.0204	
≥30, <40	0.68 (0.56-0.82)	<0.0001	0.75 (0.61-0.92)	0.0049	
≥40	0.65 (0.48-0.87)	0.0043	0.91 (0.67-1.25)	0.5768	
SOFA: ≥10, <15					
≥18.5, <25	1.0	—	1.0	—	
<18.5	1.19 (0.70-2.04)	0.5200	1.19 (0.69-2.05)	0.5380	
≥25, <30	0.89 (0.69-1.14)	0.3466	0.93 (0.72-1.20)	0.5749	
≥30, <40	0.76 (0.58-1.00)	0.0484	0.80 (0.61-1.06)	0.1191	
≥40	0.77 (0.53-1.13)	0.1849	0.95 (0.64-1.41)	0.7995	
SOFA: ≥15					
≥18.5, <25	1.0	—	1.0	—	
<18.5	0.91 (0.12-6.73)	0.9286	1.45 (0.16-13.38)	0.7433	
≥25, <30	1.17 (0.69-1.96)	0.5608	1.24 (0.67-2.30)	0.4842	
≥30, <40	0.71 (0.40-1.27)	0.2480	0.67 (0.36-1.26)	0.2124	
≥40	0.93 (0.42-2.05)	0.8544	0.83 (0.33-2.06)	0.6863	

Interaction tests and stratified analyses explored the relationship between the new BMI categories across subgroup variables. Note: adjusted for age, sex, SOFA, mechanical ventilation on the first day, renal replacement therapy on the first day, congestive heart failure, cardiac arrhythmias, valvular disease, peripheral vascular disease, hypertension, other neurological diseases, chronic pulmonary disease, liver disease, renal failure, AIDS, lymphoma, metastatic cancer, solid tumor, diabetes, fluid and electrolyte disorders, alcohol abuse, drug abuse, and depression except for the subgroup variable. Abbreviations: BMI—body mass index; HR—hazard ratio; CI—confidence interval.

## Data Availability

The raw data itself is from a third-party dataset available from MIMIC-III, a freely accessible critical care database. https://mimic.physionet.org/gettingstarted/access.

## Abbreviations

BMI: Body mass index
ICU: Intensive care unit
SOFA: Sequential organ failure assessment
MIMIC-III: Medical Information Center for Intensive Care III
SQL: Structure query language
OR: Odds ratio
HR: Hazard ratio
CI: Confidence interval
AIDS: Acquired immune deficiency syndrome
K-M: Kaplan-Meier
SID30: Elixhauser Comorbidity Index.

## Supplementary Materials

Supplementary Materials Table S1: Characteristics of normal and underweight groups. Table S2: Characteristics of normal and overweight groups. Table S3: Characteristics of normal and obese groups.
