# Supplementary material for: Association between Body Mass Index and Short-Term Clinical Outcomes in Critically Ill Patients with Sepsis: A Real-World Study
Source: Biomed Res Int. 2020 Oct 15;2020:5781913. doi: 10.1155/2020/5781913 (PMC7584974; doi:10.1155/2020/5781913)
Supplement: Supplementary Materials — Table S1: Characteristics of normal and underweight groups. Table S2: Characteristics of normal and overweight groups. Table S3: Characteristics of normal and obese groups. [file 5781913.f1.doc]

Table S1 Characteristics of normal and underweight groups

| Parameters | Normal  (≥18.5, <25 kg/m^2^) | Underweight  (< 18.5 kg/m^2^) | *P-*value |
| --- | --- | --- | --- |
| N | 2513 | 325 |  |
| Age (years) | 67.56±16.88 | 67.02±17.60 | 0.595 |
| Sex, n(%) |  |  | 0.026 |
| Male | 1363 (54.24%) | 155 (47.69%) |  |
| Female | 1150 (45.76%) | 170 (52.31%) |  |
| Mechanical ventilation on first day | 1493 (59.41%) | 175 (53.85%) | 0.055 |
| Renal replacement therapy on first day | 152 (6.05%) | 18 (5.54%) | 0.715 |
| SOFA | 5.00 (3.00-8.00) | 4.00 (3.00-8.00) | 0.007 |
| Elixhauser Comorbidity Index (SID30) | 20.00 (11.00-30.00) | 21.00 (12.00-31.00) | 0.078 |
| Length of hospital stay (days) | 13.66 (7.73-23.58) | 11.86 (7.07-21.86) | 0.032 |
| Length of ICU stay (days) | 5.23 (2.53-11.16) | 4.38 (2.22-8.99) | 0.004 |
| Hospital mortality, n(%) | 603 (24.00%) | 77 (23.69%) | 0.904 |
| ICU mortality, n(%) | 436 (17.35%) | 53 (16.31%) | 0.640 |
| 28-day mortality, n(%) | 597 (23.76%) | 89 (27.38%) | 0.151 |
| Comorbidities, n(%) |  |  |  |
| Congestive heart failure | 972 (38.68%) | 107 (32.92%) | 0.044 |
| Cardiac arrhythmias | 989 (39.36%) | 85 (26.15%) | <0.001 |
| Valvular disease | 447 (17.79%) | 49 (15.08%) | 0.226 |
| Peripheral vascular disease | 387 (15.40%) | 57 (17.54%) | 0.318 |
| Hypertension | 1286 (51.17%) | 129 (39.69%) | <0.001 |
| Other neurological disease | 387 (15.40%) | 62 (19.08%) | 0.087 |
| Chronic pulmonary disease | 602 (23.96%) | 102 (31.38%) | 0.004 |
| Liver disease | 276 (10.98%) | 39 (12.00%) | 0.583 |
| Renal failure | 523 (20.81%) | 67 (20.62%) | 0.935 |
| AIDS | 56 (2.23%) | 7 (2.15%) | 0.932 |
| Lymphoma | 68 (2.71%) | 12 (3.69%) | 0.312 |
| Metastatic cancer | 172 (6.84%) | 24 (7.38%) | 0.718 |
| Solid tumor | 129 (5.13%) | 29 (8.92%) | 0.005 |
| Diabetes | 553 (22.01%) | 45 (13.85%) | <0.001 |
| Fluid and electrolyte disorders | 1221 (48.59%) | 163 (50.15%) | 0.595 |
| Alcohol abuse | 196 (7.80%) | 14 (4.31%) | 0.024 |
| Drug abuse | 117 (4.66%) | 9 (2.77%) | 0.120 |
| Depression | 211 (8.40%) | 35 (10.77%) | 0.153 |

Data were expressed as median (Q1–Q3) or mean ± standard deviation (SD) for continuous variables and as numbers and percentages for categorical variables. Chi-square tests (for categorical variables) and Student's t test or Wilcoxon rank-sum test (for continuous variables) were used to compare differences in patient characteristics between the two groups.

**Abbreviations:** ICU, intensive care unit; SOFA, sequential organ failure assessment; AIDS, acquired immune deficiency syndrome.

Table S2 Characteristics of normal and overweight groups

| Parameters | Normal  (≥18.5, <25 kg/ m2) | Overweight  (≥25, <30 kg/ m2) | *P*-value |
| --- | --- | --- | --- |
| N | 2513 | 2465 |  |
| Age (years) | 67.56±16.88 | 66.51±16.21 | 0.026 |
| Sex, n(%) |  |  | <0.001 |
| Male | 1363 (54.24%) | 1486 (60.28%) |  |
| Female | 1150 (45.76%) | 979 (39.72%) |  |
| SOFA | 5.00 (3.00-8.00) | 6.00 (4.00-8.00) | <0.001 |
| Elixhauser Comorbidity Index (SID30) | 20.00 (11.00-30.00) | 20.00 (11.00-29.00) | 0.123 |
| Mechanical ventilation on first day | 1493 (59.41%) | 1511 (61.30%) | 0.174 |
| Renal replacement therapy on first day | 152 (6.05%) | 169 (6.86%) | 0.246 |
| Length of hospital stay (days) | 13.66 (7.73-23.58) | 14.64 (8.32-24.99) | 0.011 |
| Length of ICU stay (days) | 5.23 (2.53-11.16) | 5.86 (2.86-11.92) | 0.005 |
| Hospital mortality, n(%) | 603 (24.00%) | 533 (21.62%) | 0.046 |
| ICU mortality, n(%) | 436 (17.35%) | 397 (16.11%) | 0.240 |
| 28-day mortality, n(%) | 597 (23.76%) | 515 (20.89%) | 0.015 |
| Comorbidities, n(%) |  |  |  |
| Congestive heart failure | 972 (38.68%) | 979 (39.72%) | 0.454 |
| Cardiac arrhythmias | 989 (39.36%) | 936 (37.97%) | 0.316 |
| Valvular disease | 447 (17.79%) | 403 (16.35%) | 0.177 |
| Peripheral vascular disease | 387 (15.40%) | 365 (14.81%) | 0.559 |
| Hypertension | 1286 (51.17%) | 1332 (54.04%) | 0.043 |
| Other neurological disease | 387 (15.40%) | 369 (14.97%) | 0.672 |
| Chronic pulmonary disease | 602 (23.96%) | 529 (21.46%) | 0.036 |
| Liver disease | 276 (10.98%) | 310 (12.58%) | 0.081 |
| Renal failure | 523 (20.81%) | 588 (23.85%) | 0.010 |
| AIDS | 56 (2.23%) | 28 (1.14%) | 0.003 |
| Lymphoma | 68 (2.71%) | 72 (2.92%) | 0.646 |
| Metastatic cancer | 172 (6.84%) | 151 (6.13%) | 0.303 |
| Solid tumor | 129 (5.13%) | 112 (4.54%) | 0.332 |
| Diabetes | 553 (22.01%) | 713 (28.92%) | <0.001 |
| Fluid and electrolyte disorders | 1221 (48.59%) | 1201 (48.72%) | 0.924 |
| Alcohol abuse | 196 (7.80%) | 200 (8.11%) | 0.682 |
| Drug abuse | 117 (4.66%) | 84 (3.41%) | 0.025 |
| Depression | 211 (8.40%) | 245 (9.94%) | 0.059 |

Data were expressed as median (Q1–Q3) or mean ± standard deviation (SD) for continuous variables and as numbers and percentages for categorical variables. Chi-square tests (for categorical variables) and Student's t test or Wilcoxon rank-sum test (for continuous variables) were used to compare differences in patient characteristics between the two groups.

**Abbreviations:** ICU, intensive care unit; SOFA, sequential organ failure assessment; AIDS, acquired immune deficiency syndrome.

Table S3 Characteristics of normal and obese groups

| Parameters | Normal  (≥18.5, <25 kg/m^2^) | Obese  (≥30 kg/m^2^) | *P*-value |
| --- | --- | --- | --- |
| N | 2513 | 2664 |  |
| Age (years) | 67.56±16.88 | 63.71±14.36 | <0.001 |
| Sex, n(%) |  |  | <0.001 |
| Male | 1363 (54.24%) | 1398 (52.48%) |  |
| Female | 1150 (45.76%) | 1266 (47.52%) |  |
| SOFA | 5.00 (3.00-8.00) | 6.00 (4.00-8.00) | <0.001 |
| Elixhauser Comorbidity Index (SID30) | 20.00 (11.00-30.00) | 18.00 (9.00-28.00) | <0.001 |
| Mechanical ventilation on first day | 1493 (59.41%) | 1739 (65.28%) | <0.001 |
| Renal replacement therapy on first day | 152 (6.05%) | 197 (7.39%) | 0.154 |
| Length of hospital stay (days) | 13.66 (7.73-23.58) | 15.51 (8.91-25.39) | <0.001 |
| Length of ICU stay (days) | 5.23 (2.53-11.16) | 6.50 (3.05-13.30) | <0.001 |
| Hospital mortality, n(%) | 603 (24.00%) | 510 (19.14%) | <0.001 |
| ICU mortality, n(%) | 436 (17.35%) | 368 (13.81%) | 0.002 |
| 28-day mortality, n(%) | 597 (23.76%) | 466 (17.49%) | <0.001 |
| Comorbidities, n(%) |  |  |  |
| Congestive heart failure | 972 (38.68%) | 1136 (42.64%) | 0.010 |
| Cardiac arrhythmias | 989 (39.36%) | 1037 (38.93%) | 0.591 |
| Valvular disease | 447 (17.79%) | 418 (15.69%) | 0.119 |
| Peripheral vascular disease | 387 (15.40%) | 324 (12.16%) | 0.002 |
| Hypertension | 1286 (51.17%) | 1579 (59.27%) | <0.001 |
| Other neurological disease | 387 (15.40%) | 312 (11.71%) | <0.001 |
| Chronic pulmonary disease | 602 (23.96%) | 680 (25.53%) | 0.003 |
| Liver disease | 276 (10.98%) | 362 (13.59%) | 0.017 |
| Renal failure | 523 (20.81%) | 628 (23.57%) | 0.017 |
| AIDS | 56 (2.23%) | 13 (0.49%) | <0.001 |
| Lymphoma | 68 (2.71%) | 64 (2.40%) | 0.511 |
| Metastatic cancer | 172 (6.84%) | 89 (3.34%) | <0.001 |
| Solid tumor | 129 (5.13%) | 116 (4.35%) | 0.388 |
| Diabetes | 553 (22.01%) | 1157 (43.43%) | <0.001 |
| Fluid and electrolyte disorders | 1221 (48.59%) | 1291 (48.46%) | 0.983 |
| Alcohol abuse | 196 (7.80%) | 215 (8.07%) | 0.906 |
| Drug abuse | 117 (4.66%) | 81 (3.04%) | 0.006 |
| Depression | 211 (8.40%) | 300 (11.26%) | 0.003 |

Data were expressed as median (Q1–Q3) or mean ± standard deviation (SD) for continuous variables and as numbers and percentages for categorical variables. Chi-square tests (for categorical variables) and Student's t test or Wilcoxon rank-sum test (for continuous variables) were used to compare differences in patient characteristics between the two groups.

**Abbreviations:** ICU, intensive care unit; SOFA, sequential organ failure assessment; AIDS, acquired immune deficiency syndrome.
